# Opportunistic osteoporosis screening: contrast-enhanced dual-layer spectral CT provides accurate measurements of vertebral bone mineral density

**DOI:** 10.1007/s00330-020-07319-1

**Published:** 2020-10-14

**Authors:** Ferdinand Roski, Johannes Hammel, Kai Mei, Bernhard Haller, Thomas Baum, Jan S. Kirschke, Daniela Pfeiffer, Klaus Woertler, Franz Pfeiffer, Peter B. Noël, Alexandra S. Gersing, Benedikt J. Schwaiger

**Affiliations:** 1 Department of Radiology, Klinikum rechts der Isar, School of Medicine, Technical University of Munich, Ismaninger Str. 22, 81675 Munich, Germany; 2 Biomedical Physics & Munich School of BioEngineering, Technical University of Munich, 85748 Garching, Germany; 3 Department of Radiology, Perelman School of Medicine, University of Pennsylvania, Philadelphia, PA 19104 USA; 4 Institute of Medical Statistics and Epidemiology, Klinikum rechts der Isar, School of Medicine, Technical University of Munich, 81675 Munich, Germany; 5 Department of Neuroradiology, Klinikum rechts der Isar, School of Medicine, Technical University of Munich, 81675 Munich, Germany

**Keywords:** Bone density, Contrast media, Osteoporosis, Tomography, X-ray computed

## Abstract

**Objectives:**

Osteoporosis remains under-diagnosed, which may be improved by opportunistic bone mineral density (BMD) measurements on CT. However, correcting for the influence of intravenous iodine-based contrast agent is challenging. The purpose of this study was to assess the diagnostic accuracy of iodine-corrected vertebral BMD measurements derived from non-dedicated contrast-enhanced phantomless dual-layer spectral CT (DLCT) examinations.

**Methods:**

Vertebral volumetric DLCT-BMD was measured in native, arterial, and portal-venous scans of 132 patients (63 ± 16 years; 32% women) using virtual monoenergetic images (50 and 200 keV). For comparison, conventional BMD was determined using an asynchronous QCT calibration. Additionally, iodine densities were measured in the abdominal aorta (AA), inferior vena cava, and vena portae (VP) on each CT phase to adjust for iodine-related measurement errors in multivariable linear regressions and a generalized estimated equation, and conversion equations were calculated.

**Results:**

BMD values derived from contrast-enhanced phases using conversion equations adjusted for individual vessel iodine concentrations of VP and/or AA showed a high agreement with those from non-enhanced scans in Bland-Altman plots. Mean absolute errors (MAE) of DLCT-BMD were 3.57 mg/ml for the arterial (*R*^2^ = 0.989) and 3.69 mg/ml for the portal-venous phase (*R*^2^ = 0.987) (conventional BMD: 4.70 [*R*^2^ = 0.983] and 5.15 mg/ml [R^2^ = 0.981]). In the phase-independent analysis, MAE was 4.49 mg/ml for DLCT (*R*^2^ = 0.989) (conventional BMD: 4.82 mg/ml [*R*^2^ = 0.981]).

**Conclusions:**

Converted BMD derived from contrast-enhanced DLCT examinations and adjusted for individual vessel iodine concentrations showed a high agreement with non-enhanced DLCT-BMD, suggesting that opportunistic BMD measurements are feasible even in non-dedicated contrast-enhanced DLCT examinations.

**Key Points:**

*• Accurate BMD values can be converted from contrast-enhanced DLCT scans, independent from the used scan phase.*

*• DLCT-BMD measurements from contrast-enhanced scans should be adjusted with iodine concentrations of portal vein and/or abdominal aorta, which significantly improves the goodness-of-fit of conversion models.*

## Introduction

Osteopenia and osteoporosis remain a severe challenge in health care, not only from a clinical perspective—the treatment gap is estimated at 59% of women and 57% of men in the EU [1]—but also from an epidemiologic view considering its contribution to an increasing number of fall-related deaths [2]. Bearing in mind that the majority of the causal vertebral fragility fractures (VFFs) already occurs in osteopenic individuals [3] emphasizes the importance of early detecting patients at risk. A UK audit, however, showed that incidental VFFs are substantially underdiagnosed in non-dedicated CT exams due to deficient reporting [4]. Here, even basic tools (e.g., routine sagittal reformations, stringent terminology) could improve clinico-radiological workflow and patients’ outcome [5]. Besides, fracture-related treatment costs represent the main cost factor for osteoporosis, thus causing considerable economic burden to health systems [1].

A diagnostic gap was not only found in patients with primary or postmenopausal osteoporosis [6–8]—this is also an issue regarding patients with secondary causes for reduced bone mineral density (BMD), e.g., patients suffering from malignant conditions and undergoing therapy also affecting BMD [9]. For instance, men with prostate cancer receiving androgen deprivation therapy (ADT) bear an increased risk to suffer from accelerated bone loss, which is a major adverse effect [10–12]. Even though guidelines recommend evaluation with baseline and periodic follow-up BMD quantification [13], there is a severe under-use of dedicated imaging methods such as dual-energy x-ray absorptiometry (DXA) or quantitative CT (QCT)—e.g., for patients with non-metastatic prostate cancer, only about one in ten patients over 65 years receiving ADT undergoes a baseline bone densitometry [14–16].

Most patients with such malignant conditions are regularly subjected to contrast-enhanced CT for (re-)staging or the assessment of age-related comorbidities, in which a vast set of imaging data is generated. This gave rise to the question whether contrast-enhanced CT scans may be used for opportunistic BMD measurements to avoid additional dedicated examinations.

In clinical osteodensitometry, dual-energy CT (DECT) imaging has been available for more than 30 years [17]. However, in contrast to other DECT approaches such as dual-source CT or single-source CT with rapid kV switching, which are routinely performed in single-energy mode, DLCT continuously detects dual-energy information in standard CT protocols. Consequently, this novel implementation of DECT provides spectral data on clinical demand in all examinations without the need to prospectively select qualified patients for DECT imaging. Providing spectral information retrospectively in all examinations not only does facilitate radiological workflow, but also virtually lends itself for the retrospective approach of opportunistically measuring BMD.

By means of two superimposed detector layers, dual-layer spectral CT (DLCT) enables the separation of low- and high-energy photons, therefore providing energy-specific attenuation coefficients of present materials [18]. Based on this spectral information, one can infer on the material composition of body tissues or fluids, e.g., by measuring the concentration of calcium hydroxyapatite for BMD quantification [19] or the iodine concentration for quantifying contrast enhancement [20].

In a previous publication, the feasibility of opportunistic screening for osteoporosis was already demonstrated in vivo for native DLCT examinations [21]. Consequently, the scope of applicability in this paper is confined to clinical settings in which current non-enhanced DLCT data are missing.

The objective of this study was to evaluate the usability of contrast-enhanced DLCT scans for calculating precise BMD values based on non-dedicated CT examinations, i.e., with examinations performed for indications other than osteodensitometry.

For this purpose, the present analysis investigated (I) the effects of iodine contrast agent on the accuracy of DLCT-BMD measurements and (II) any possible improvement of accuracy by adjusting for concentrations of iodine contrast agent measured in large vessels.

## Materials and methods

### Patient population

According to the study protocol, 471 consecutive patients with a complete triphasic DLCT examination of the abdomen or thorax/abdomen were enrolled if exams were performed for indications other than osteodensitometry. Of those, patients with metal-containing implants adjacent to the thoracolumbar spine such as aortic stent grafts (*n* = 293) or spinal instrumentation (*n* = 36) were excluded. The same applies for patients with malignant conditions affecting the spine, e.g., spinal bone metastases (*n* = 7) or hematologic diseases (*n* = 3), resulting in a patient sample of 132 patients (63 ± 16 years; 32% women) who were retrospectively identified in the institutional PACS between September 2016 and October 2018.

Institutional Review Board approval was obtained prior to this study (Ethics Commission of the School of Medicine, Technical University of Munich, Germany). Informed consent was waived for this retrospective analysis of routinely acquired imaging data.

### DLCT image acquisition

CT images were acquired with one DLCT scanner (IQon Spectral CT, Philips Healthcare). For all scans, a routine abdominal CT protocol was used with a tube voltage of 120 keV. Non-contrast-enhanced (NE) scans had an exposure of 107 ± 49 mAs (mean ± SD) and a mean CT dose index (CTDI_vol_) of 9.7 ± 4.5 mGy; contrast-enhanced (CE) scans had an exposure of 110 ± 43 mAs and a mean CTDI_vol_ of 10.0 ± 3.9 mGy.

CE scans were performed using iomeprol, a non-ionic iodinated contrast agent for intravenous application (Imeron 400 MCT, Bracco Imaging Deutschland GmbH). Per routine clinical protocol and depending on the clinical situation, a volume of 50 to 70 ml was administered with a flow rate of 3 ml/s. Arterial phases were triggered when the average CT numbers of a volume of interest (VOI) in the descending thoracic aorta exceeded a threshold value of 150 HU. The portal-venous phases started 70 s after contrast administration.

### Image analysis, post-processing, and BMD calculation

**Fig. 1:**
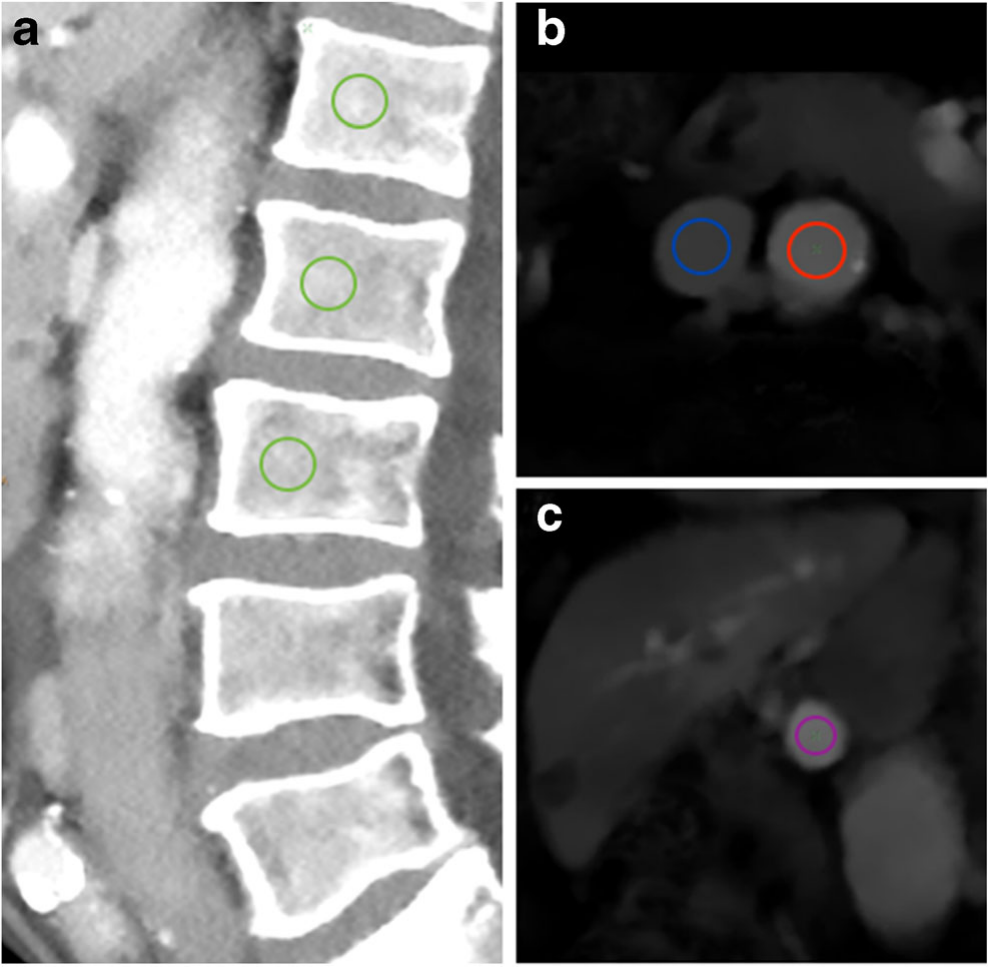
Sagittal (**a**), axial (**b**), and multiplanar reformation (**c**) of dual-layer spectral CT imaging of a 77-year-old male patient. Spinal VOIs are positioned in the ventral halves of L1–L3 (green) (VMI at 50 keV). Vascular VOIs are positioned orthogonally and mid-luminally in the abdominal aorta (red), the inferior vena cava (blue), and the portal vein (violet) (iodine density maps)

On sagittal reformations, circular VOIs were manually placed in the ventral halves of L1 to L3. If one or more of these showed a pathology such as a fracture or extensive degenerative changes, adjacent thoracolumbar vertebrae were used instead. CT numbers in NE, arterial (AR), and portal-venous (PV) phases were extracted from both conventional and two virtual monoenergetic images at different energy levels (VMI; 50 and 200 keV), respectively (Fig. 1).

Subsequently, patients’ BMD values were calculated in two ways: Based on VMIs, DLCT-BMD values were computed for all three scan phases as previously described by Roski et al [21]. In short, after performing an ex vivo high-dose calibration scan of three hydroxyapatite (HA)-containing insets of the European spine phantom (ESP) with DLCT, attenuation values of VMIs can be linearly assigned to respective BMD values via projection calibration as they are proportional to measured HA concentrations.

Conventional BMD values were similarly calculated for all scan phases by using an asynchronous calibration [22, 23] with attenuation values of a standard QCT phantom (Mindways QCT) consisting of five differently concentrated solutions of K_2_PO_4_. Here, phantom measurements were averaged over 33 examinations, which were performed in the same time period and on the same scanner. Instead of VMIs, conventional images were used for BMD quantification. These are reconstructed by using a weighted sum of counts measured in the two detector layers, with resulting images being analogous to a conventional single-energy CT scanner [24].

Additionally, mid-luminal iodine concentrations (mg/ml) were measured within the abdominal aorta (AA) and the inferior vena cava (IVC) on axial images—immediately inferior to the renal vessels—as well as within the vena portae (VP) on multiplanar reformations (Fig. 1). A commercially available spectral CT software was used for the generation of iodine density maps based on iodine-water decomposition (IntelliSpace Portal 10.1.0, Philips Healthcare).

### Statistical analysis

Both DLCT and conventional BMD values from CE scans were separately correlated with their reference values from corresponding NE scans in multivariable linear regression models using forward selection. BMD values from CE scans, vessel iodine concentrations (AA, IVC, and VP), age, and sex represented the set of selectable independent variables. DLCT-BMD from NE scans had previously shown its validity in a comparison with QCT [21]. Consequently, these data served as standard of reference for the contrast-enhanced DLCT-BMD values in this study. Likewise, the respective native values served as the dependent variable for conventional BMD. This linear regression analysis was performed separately for AR and PV. For the phase-independent (PI) approach, due to present within-subject correlation, generalized estimated equations (GEE) were used to investigate functional relations between contrast-enhanced and native BMD.

Beforehand, the patient sample was randomly split up into a training cohort (*n* = 88) for the multivariable linear regression model and a test cohort (*n* = 44) for the eventual validation of its predictive accuracy. For the phase-independent GEE analysis, individual patient scans of the AR and PV phase were independently assigned to the training cohort (*n* = 176) and the test cohort (*n* = 88).

Bland-Altman plots showing data from the test cohorts were used to examine the agreement of native and calculated BMD values, thus estimating the predictive value of the regression model. Moreover, agreement was calculated on a patient base as mean of absolute errors (MAEs) before and after the application of conversion equations. The external data of the test cohort was used to validate the conversion equations of both linear regressions and the GEE analysis with *R*^2^ values.

The statistical calculations were performed using SPSS 23 (IBM).

## Results

### In vivo DLCT-based and conventional BMD measurements

**Fig. 2:**
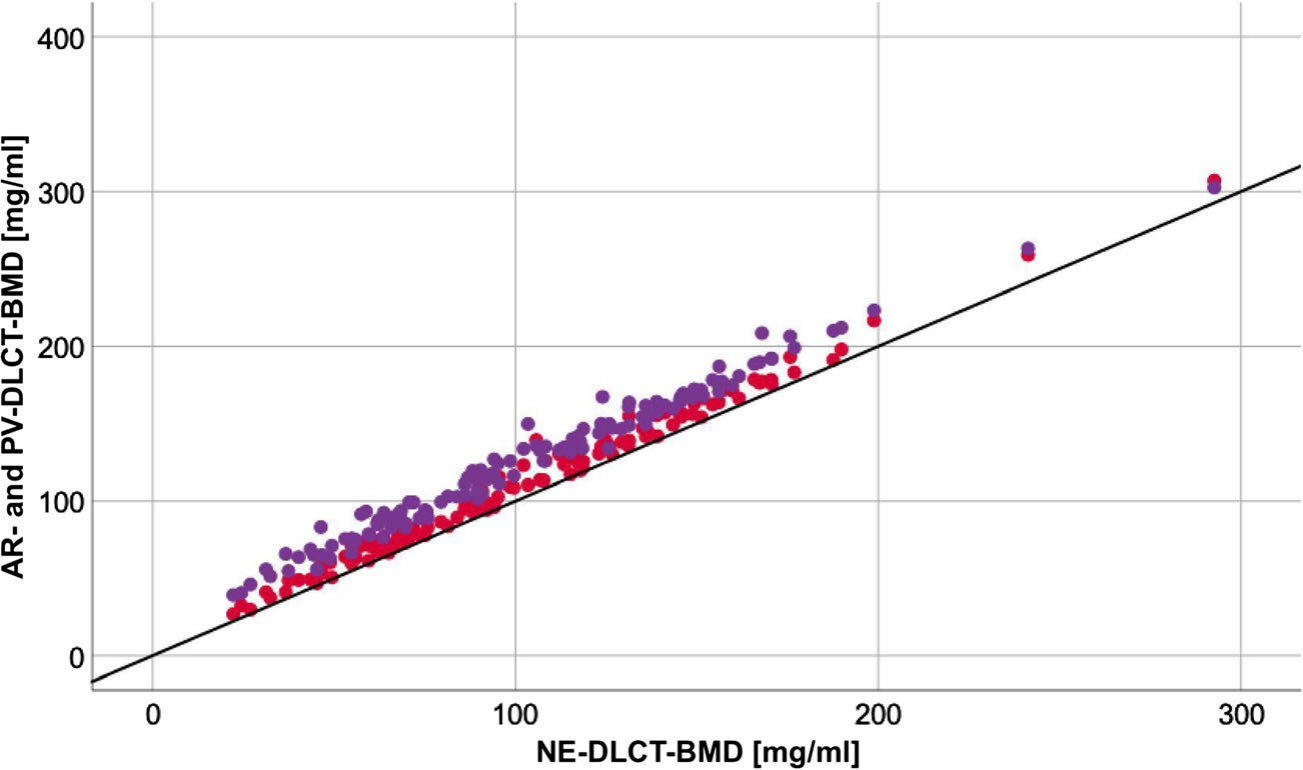
Scatter plot showing DLCT-BMD values of 132 patients (averaged over three vertebrae each) derived from different contrast-enhanced scan phases (AR = red and PV = violet); the black bisecting line serves as standard of reference showing the corresponding line of native BMD values

For DLCT-BMD, measurements in native scans averaged to 102.95 ± 46.33 mg/ml (mean ± SD), whereas arterial (112.23 ± 7.88 mg/ml [+ 9.0%]) and portal-venous (126.86 ± 53.89 mg/ml [+ 23.2%]) scan phases revealed substantially higher results (Fig. 2). DLCT-BMD from AR and PV showed high correlations (*r* = 0.994 [95% confidence interval, 0.991–0.996] and *r* = 0.989 [0.984–0.992]), yet a low agreement with DLCT-BMD from NE.

Conventionally calculated BMD showed similar, yet continuously lower results: native BMD values were 97.00 ± 43.48 mg/ml, whereas in CE phases, likewise, BMD values were substantially higher with 106.44 ± 44.35 mg/ml (+ 9.7%) for AR and 119.67 ± 42.93 mg/ml (+ 23.3%) for PV, respectively.

### Linear regression and generalized estimating equation analyses

Both phase-specific regression models had very high coefficients of determination for DLCT-BMD measurements with conventional BMD values consistently showing lower goodness of fit (Table 1).

**Table 1:** Comparison of *R*^2^ change and adjusted *R*^2^ for several multivariable linear regression analyses with forward selection, either based on contrast-enhanced DLCT-BMD or on conventional BMD data, respective native BMD values served as dependent variable, illustrated for AR and PV

	Variable	DLCT-BMD (*R*^2^c/*R*^2^adj)	Conv. BMD (*R*^2^c/*R*^2^adj)
a) arterial phase			
AR			
1	BMD_AR_	0.983 (*p* = 0.000)	0.978 (*p* = 0.000)
2	Iodine_VP_	0.007 (*p* = 0.000)	0.008 (*p* = 0.000)
Total		0.990	0.986
b) portal-venous phase			
PV			
1	BMD_PV_	0.976 (*p* = 0.000)	0.966 (*p* = 0.000)
2	Iodine_AA_	0.008 (*p* = 0.000)	0.010 (*p* = 0.000)
3	Iodine_VP_	0.001 (*p* = 0.019)	-
Total		0.984	0.975

Besides respective BMD values from CE scan phases, a significant association of the iodine concentration of the VP could be detected for all three DLCT models (AR: change of *R*^2^ (*R*^2^c) = 0.007 (DLCT-BMD)/*R*^2^c = 0.008 (conventional BMD), PV: *R*^2^c = 0.001/not significant). Patient’s age was significantly associated with the outcome only for the phase-independent GEE analysis, the iodine concentration of AA only for the PV linear regression model (*R*^2^c = 0.008/*R*^2^c = 0.010). Neither iodine concentration of IVC nor sex was identified as a significant predictor for any scan phase.

The derived conversion equations for the DLCT-BMD data are:

$$ {\displaystyle \begin{array}{c}\mathrm{cBMD}\left(\mathrm{AR}\right)=0.989\times \mathrm{BMD}\left(\mathrm{AR}\right)-2.170\times \mathrm{Iodine}\left(\mathrm{VP}\right)-4.070\\ {}\begin{array}{c}\mathrm{cBMD}\left(\mathrm{PV}\right)=0.981\times \mathrm{BMD}\left(\mathrm{PV}\right)-1.407\times \mathrm{Iodine}\left(\mathrm{AA}\right)-1.109\times \mathrm{Iodine}\ \left(\mathrm{VP}\right)-5.928\\ {}\mathrm{cBMD}\left(\mathrm{PI}\right)=0.941\times \mathrm{BMD}\left(\mathrm{CE}\right)-2.636\times \mathrm{Iodine}\left(\mathrm{VP}\right)-0.141\times \mathrm{Age}+9.233\end{array}\end{array}} $$

### Agreement of native and calculated BMD

**Fig. 3:**
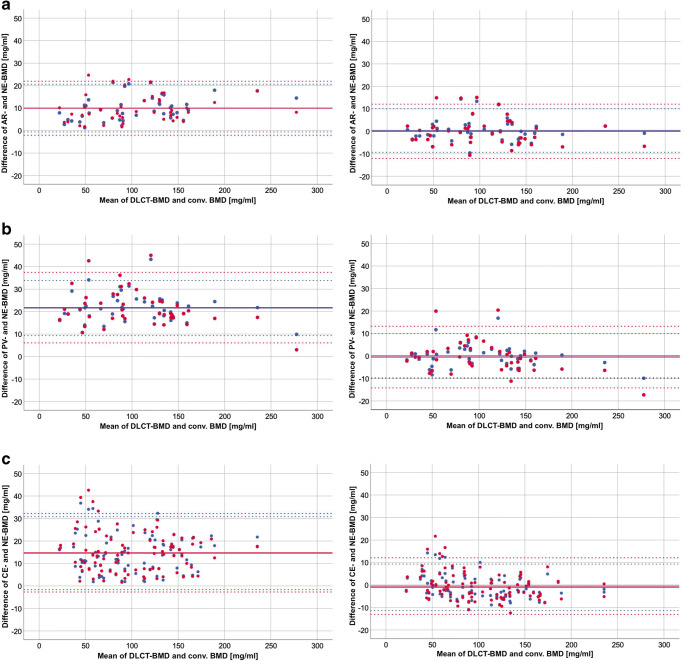
Bland-Altman plots showing data of randomly selected test cohorts (*n* = 44 for AR and PV, *n* = 88 for PI); the *y*-axis shows differences between BMD derived from contrast-enhanced scan phases and native BMD; the *x*-axis shows mean values of native DLCT-BMD and native conventional BMD; the black solid lines indicate equivalence of BMD values; the colored solid lines indicate the mean of BMD differences; the colored dotted lines indicate the 95% limits of agreement (mean difference ± 1.96 SD); blue represents data of DLCT-BMD; red represents data of conventional BMD; top row = AR (**a**), middle row = PV (**b**), bottom row = PI (**c**); left column: BMD data before application of conversion equation, right column: estimated BMD after application of conversion equation

After converting the BMD values of the test cohorts using the presented equations, corresponding Bland-Altman plots showed a substantial agreement between converted and respective natively measured BMD (Fig. 3).

For the arterial phase, DLCT-BMD had a mean of absolute errors (MAEs) of 9.99 (95% confidence interval, 8.38–11.59) mg/ml before and 3.57 (2.56–4.58) mg/ml after the conversion of measured to estimated BMD, whereas conventional BMD improved from 9.93 (8.12–11.75) to 4.70 (3.53–5.86) mg/ml.

For the portal-venous phase, conversion of contrast-enhanced data reduced MAE from 21.58 (19.73–23.42) mg/ml to 3.69 (2.69–4.70) mg/ml for DLCT-BMD (21.76 [19.39–24.13] to 5.15 [3.76–6.54] mg/ml for conventional BMD).

The MAE of the phase-independent GEE model changed from 14.57 (12.81–16.33) mg/ml before to 4.49 (3.87–5.11) mg/ml after the conversion of DLCT-BMD (14.77 [12.88–16.66] to 4.82 [3.99–5.64] mg/ml for conventional BMD).

The above conversion equations were validated with external data of the test cohorts and consistently showed high coefficients of determination (Table 2).

**Table 2:** Coefficients of determination (*R*^2^) for external data of the test cohorts (*n* = 44 for AR and PV, *n* = 88 for PI)

	DLCT-BMD (*R*^2^)	Conv. BMD (*R*^2^)
Arterial phase (AR)	0.989	0.983
Portal-venous phase (PV)	0.987	0.981
Phase-independent analysis (PI)	0.989	0.981

## Discussion

Converted BMD values, derived from routine clinical contrast-enhanced DLCT and adjusted for vessel iodine concentrations, showed a high agreement with non-enhanced DLCT-BMD. Moreover, the phase-independent conversion equation provides results which are adequate for the detection of low BMD in a clinical context as well.

Before this conversion, DLCT-BMD values were consistently higher for all scan phases when measured in contrast-enhanced scans compared with their native BMD references (Fig. 2). This represents the challenge of adequately separating intravascular iodine within the vertebra from HA, which is attributable to similar spectral absorption behavior of the two components. Another explanation for this BMD variation is the present dual-layer set-up, which cannot provide absolute selectivity on the detector level due to an overlap of the high- and low-energy spectra [25, 26].

As the very same VOIs were used for comparing both calculations, the utilization of spectral information is capable of additionally improving overall BMD accuracy. Although DLCT is still not perfectly specific for HA, the consistently higher coefficients of determination in the linear regression (Table 1) and the GEE analysis suggest that DLCT-BMD shows a more pronounced functional relation between contrast-enhanced and native scans compared with conventional BMD. Besides, quantifying iodine concentrations outside osseous structures, more precisely in large vessels (Vena portae, Aorta), significantly improves the accuracy of converted BMD.

In a pilot analysis (*n* = 12), tissue iodine concentrations of paraspinal muscle and fat were measured for all scan phases. Balancing the linear model between completeness and complexity, however, the rationale was to set up a slender, numerically stable model without overfitting or multicollinearity. Here, preliminary data suggested focusing on vessel iodine measurements in order to adjust for the influence of intravascular contrast agent. After the conversion, DLCT-BMD showed a high agreement between native and converted values in Bland-Altman plots (Fig. 3a–c) and a substantial reduction of MAEs (relative reduction of 64% for AR, 83% for PV, and 69% for PI). The validation with external data of the test cohort revealed coefficients of determination that are equivalent to those of the training cohort (Table 2). This finding confirms a numerically stable model for BMD prediction that is accurate in new data.

Particularly the phase-independent approach features critical advantages over phase-specific conversion equations as in clinical routine, the iodine concentration within the vertebra’s trabecular compartment may be affected by numerous factors apart from scan timing [27, 28]: e.g., contrast application speed, volume, the patient’s circulatory capacity, volume of distribution, red vs. yellow bone marrow ratio. BMD values derived from a phase-independent conversion equation could minimize the influence of those factors and therefore may be most useful in clinical reality.

Focusing on contrast-enhanced clinical DLCT exams, these routinely acquired clinical CT data were utilized for opportunistic BMD measurements to monitor changes such as tumor- or therapy-associated bone loss, when current non-enhanced scans are missing. The proposed DLCT-based method provides the opportunity to retrospectively screen for low BMD and could at once spare patients additional exposure to radiation by dedicated bone densitometric exams. Quantifying individual iodine concentrations in abdominal vessels turned out to be a practicable way of adjusting for the influence of intravascular contrast agent. In this context, there is extensive literature indicating the high accuracy of DLCT-based iodine quantification: within the typically encountered concentration ranges in clinical radiology, relative mean errors of about 3.3 to 4.6% were found, with simulated patient size and tube voltage inconsistently affecting measuring precision [20, 29, 30].

Comparable studies assessing iodine-associated effects on BMD quantification with multidetector CT (MDCT) encountered limitations: a study by Baum et al had a small training cohort for the conversion equation, relied on phantom calibration and only used PV scans [31]. A comparable study by Kaesmacher et al was limited by a small number of enrolled patients [32].

The conversion equations in this DLCT study, however, are based on a solid training cohort of 88 patients. An additional correction step was introduced by adjusting for vascular iodine concentrations, which can be obtained with minimal effort in work and time. Besides, DLCT potentially combines the inherent benefits of dual-energy imaging for osteodensitometry with the major advantage of QCT, i.e., exclusive volumetric measurements of the trabecular compartment, which is more sensitive regarding therapy-associated bone remodeling processes [33]—however, without needing synchronous phantom calibration.

Note that the present statistical models already show very high determination coefficients for conventional BMD: contrary to a comparable study investigating asynchronously calibrated BMD derived from contrast-enhanced MDCT, DLCT-BMD has substantially narrower 95% limits of agreement (− 10 to + 11 mg/ml (DLCT) vs. ca. − 30 to + 14 mg/ml (MDCT) for AR and − 10 to + 10 mg/ml (DLCT) vs. ca. − 39 to + 8 mg/ml for PV) and a better linear fit (*R*^2^: 0.983 vs. 0.923 for AR, 0.976 vs. 0.904 for PV) [32]. Considering the minor contribution of the vessel iodine corrections, these results suggest a notably higher measuring accuracy of the DLCT scanner compared with MDCT.

Apart from several phantom studies, two in vivo trials already showed the diagnostic accuracy of native DLCT regarding osteodensitometric applications: Van Hedent et al demonstrated that DLCT-based BMD measurements perform very well in the detection of decreased BMD using DXA as standard of reference [34]. A previously mentioned study by Roski et al showed that non-enhanced DLCT-based BMD measurements are on a par with phantom-based QCT [21].

This study has limitations. As CT exams were retrieved from clinical routine, there was no systematic variation in the amount of applied contrast agent to adjust for contrast load. Furthermore, neither overall circulatory parameters nor the local vascularization of the vertebral bodies for correlating contrast distribution could be investigated according to the retrospective nature of this analysis. Moderating both scan protocol inconsistencies and varying circulatory parameters, the phase-independent analysis is potentially meeting clinical reality best. Besides, the vertebral VOIs were placed manually, which contributes to the risk of a higher intra- or interobserver variability. A next step would be to overcome this issue by implementing a BMD analysis pipeline drawing on automatic segmentation. Additional longitudinal studies will be needed to investigate the in vivo reproducibility and the predictive power regarding incidental fractures.

In summary, this study showed that BMD values can be accurately estimated from contrast-enhanced multiphasic dual-layer spectral CT examinations, even independently from the used contrast phase. Moreover, measuring only one abdominal vessel for iodine concentration could significantly increase the goodness-of-fit in statistical models. Therefore, iodine-adjusted DLCT-BMD measurements suggest their potential value for a reliable opportunistic assessment of BMD even in routine clinical contrast-enhanced examinations.

## Abbreviations

AA: Aorta abdominalis
ADT: Androgen deprivation therapy
AR: Arterial scan phase
BMD: Bone mineral density
cBMD: Converted BMD
CE: Contrast-enhanced
CTDI_vol_: CT dose index
DECT: Dual-energy computed tomography
DLCT: Dual-layer spectral computed tomography
DXA: Dual-energy x-ray absorptiometry
GEE: Generalized estimated equations
HA: Calcium hydroxyapatite
IVC: Inferior vena cava
MAE: Mean absolute error
MDCT: Multidetector computed tomography
PI: Phase-independent
PV: Portal-venous scan phase
QCT: Quantitative computed tomography
R^2^c: Change of *R*^2^
VFFs: Vertebral fragility fractures
VMI: Virtual monoenergetic image
VOI: Volume of interest
VP: Vena portae
